# Cloning and disruption of the *UeArginase* in *Ustilago esculenta*: evidence for a role of arginine in its dimorphic transition

**DOI:** 10.1186/s12866-019-1588-2

**Published:** 2019-09-05

**Authors:** Yafen Zhang, Min Wu, Qianwen Ge, Mengfei Yang, Wenqiang Xia, Haifeng Cui, Xiaoping Yu, Shangfa Zhang, Zihong Ye

**Affiliations:** 1 Zhejiang Provincial Key Laboratory of Biometrology and Inspection & Quarantine, College of Life Sciences, China Jiliang University, Hangzhou, 310018 Zhejiang China; 2 Jinhua Academy of Agricultural Sciences, Jinhua, Zhejiang China

**Keywords:** *Ustilago esculenta*, *UeArginase*, Arginine metabolism, Dimorphic transition

## Abstract

**Background:**

*Ustilago esculenta*, a typical dimorphic fungus could infect *Zizania latifolia* and induce host stem swollen to form an edible vegetable called Jiaobai in China. The strains differentiation especially in the mating ability and pathogenicity is closely related to different phenotypes of Jiaobai formed in the fields. Dimorphic switching, a tightly regulated processes, is essential for the pathogenetic development of dimorphic fungi. In responses to environment cues, dimorphic switching can be activated through two conserved cell signaling pathways-PKA and MAPK pathways. Previous study indicated that exogenous arginine could induce hyphal formation in several dimorphic fungi through hydrolysis by arginase, but inhibit the dimorphic transition of *U. esculenta*. We conducted this study to reveal the function of arginine on dimorphic transition of *U. esculenta*.

**Results:**

In this study, we found that arginine, but not its anabolites, could slow down the dimorphic transition of *U. esculenta* proportionally to the concentration of arginine. Besides, *UeArginase*, predicated coding arginase in *U. esculenta* was cloned and characterized. *UeArginase* mutants could actually increase the content of endogenous arginine, and slow down the dimorphic transition on either nutritious rich or poor medium. Either adding exogenous arginine or *UeArginase* deletion lead to down regulated expressions of *UePkaC*, *UePrf1*, *mfa1.2*, *mfa2.1*, *pra1* and *pra2*, along with an increased content of arginine during mating process.

**Conclusion:**

Results of this study indicated a direct role of arginine itself on the inhibition of dimorphic transition of *U. esculenta*, independent of its hydrolysis by UeArginase.

**Electronic supplementary material:**

The online version of this article (10.1186/s12866-019-1588-2) contains supplementary material, which is available to authorized users.

## Background

Dimorphic switching, known as a tightly regulated processes that switching between hyphal growth and yeast-like state, is essential for the pathogenesis of both animal and plant pathogenic dimorphic fungi [1]. There are many environment cues have been described to be important inducers in the dimorphic switching, such as carbon or nitrogen sources [2, 3], temperature [4], pH [5], growth atmosphere [6], or host signals [5]. However, the cell signaling pathway networks in dimorphic fungi are conserved [5]. The cAMP-dependent protein kinase A (PKA) pathway and the mitogen-activated protein kinase (MAPK) pathway have been proved at the heart of this network, responding to the environment cues to regulate fungal dimorphism [1]. Most fungi exhibit dimorphism in response to distinct carbon or nitrogen sources [3], e. g., in *Mucor* species, yeast growth is preferred when a fermentable hexose is available [7]. Studies showed that arginine, as a nitrogen source, could induce hyphal formation in several dimorphic fungi [8, 9], e. g., 10 mM exogenous arginine could induce the dimorphic switching from yeast to mycelia in *Ceratocystis ulmi* to cause elm disease [8]; for *Candida albicans*, an increase in arginine through exogenous addition or endogenous synthesis, could induce germ tube formation to escape from macrophage in a density-dependent manner [9]. Further researches have proved that the metabolites of arginine activate adenylyl cyclase to synthesize cAMP, which in turn activates PKA pathway to trigger the morphogenetic switch from yeast to hyphae in *C. albicans* [9].

In the typical smut fungus *U. maydis*, dimorphism is a particular irreversible growth form that switching from a haploid, unicellular phase to a dikaryotic filamentous stage [1]. It implies a critical role of mating (including cell fusion and hyphal growth in the life cycle) in pathogenetic development [10]. The *a* genes and *Prf1* gene are responsible for mating [11–13], which is regulated by some MAPK pathway members such as Kpp2 and Kpp6 [14], and the cAMP-PKA pathway, in which Adr1 and Ubc1 are involved [15]. Acidic pH [5], pheromone [16], starvation of nitrogen [17], fatty acids as carbon source [18] and plant signals such as hydrophobicity [19] have been discovered to influence the mating process in *U. maydis*. However, there is no report of arginine involved in dimorphism of *U. maydis* and other smut fungi.

*Ustilago esculenta*, resembling an endophytic smut fungus in a perennial root herb plant *Zizania latifolia* [20, 21], would inhibit host flowering and induce host stem swollen to form a flavored vegetable in Southeast Asian [22, 23]. Similarly to *U. maydis*, *U. esculenta* undergoes a dimorphism process from a saprophytic yeast-like haploid stage to a pathogenic heterokaryotic mycelial stage [24, 25]. Evidences showed that dimorphism transition of *U. esculenta* was started after pheromone-receptor recognition and followed by conjugation tubes formation and cells fusion [26]. This process was determined by compatible *a* genes and regulated by UePrf1, which can interact with UeKpp2 and UeKpp6 [21, 26, 27]. Besides, environmental signals such as changes of carbon and nitrogen sources, pH can influence dimorphism of *U. esculenta* through MAPK signaling cascades [25, 28]. Fortunately, a completely sexual cycle of *U. esculenta* has been carried out and the yeast-to-mycelium dimorphic transition of *U. esculenta* can be obtained in vitro in our laboratory [25]. Based on the whole genome sequence analysis [21], it is supposed that, like *U. maydis* and other fungi [29], *U. esculenta* can utilize arginine as an alternative nitrogen source, through hydrolysis into ornithine and urea, which is catalyzed by arginase. In this study, we found that excess arginine inhibited the dimorphic transition of *U. esculenta*. Besides, a homologue gene coding arginase named *UeArginase* in *U. esculenta* was cloned and characterized. Similar to adding exogenous arginine, mutation of *UeArginase* could actually increase the content of endogenous arginine, and slow down the mating process, along with down regulated expressions of *UePkaC*, *UePrf1*, *mfa1.2*, *mfa2.1*, *pra1* and *pra2*. These findings are supplements of the function of arginine on the fungal dimorphic transition, and are important for studying the interaction between *U. esculenta* and *Z. latifolia*. However, the mechanism of arginine-mediated dimorphic switching in *U. esculenta* still needs further researches.

## Results

### Exogenous arginine decelerates dimorphic transition

**Fig. 1:**
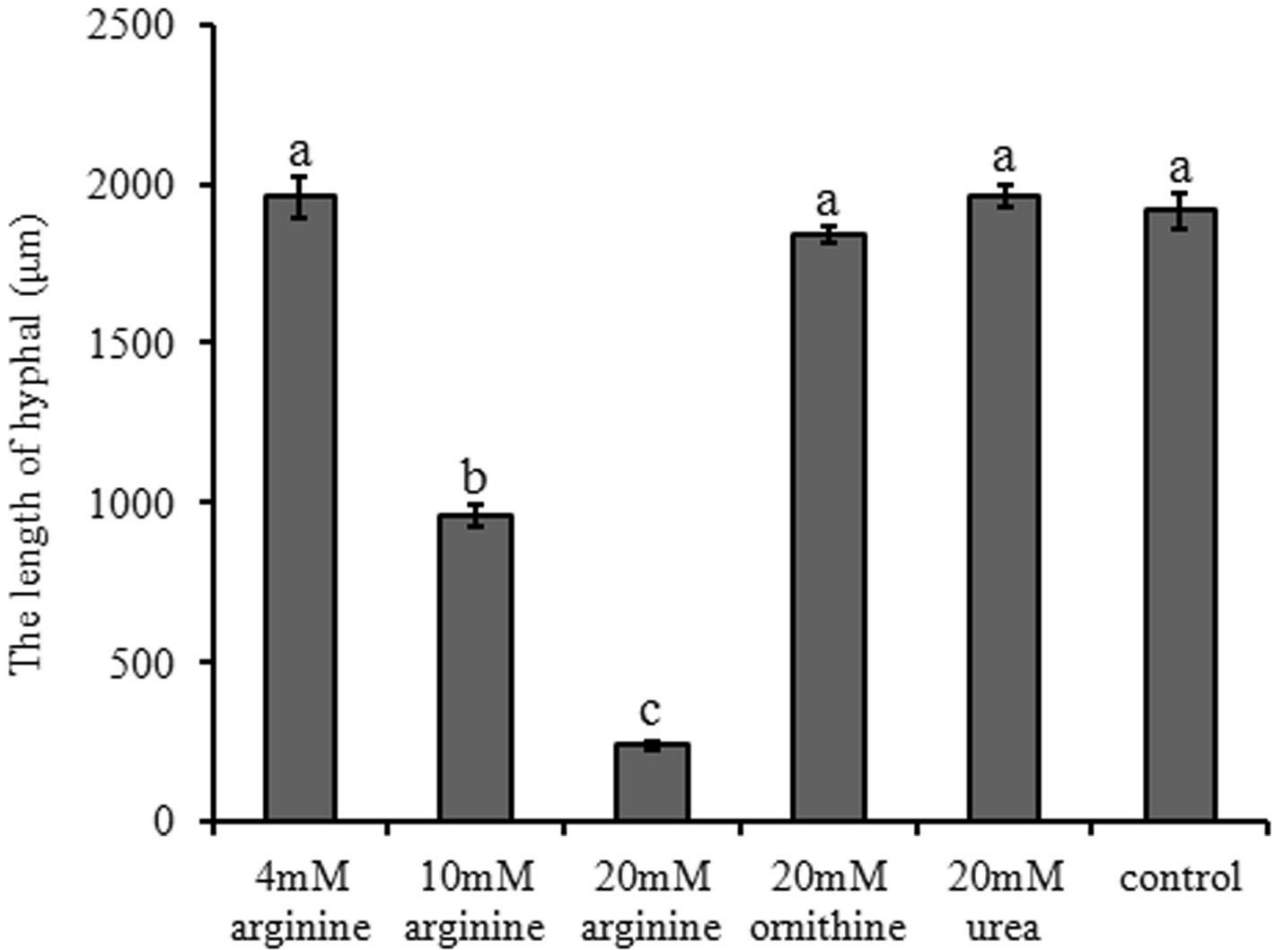
The influence of arginine and its anabolites on the hyphal growth of *U. esculenta*. The hyphal length of the mated colonies under specified medium (X-axis) after 3 days culturing were measured under stereomicroscope. Differences in hyphal length were analyzed by One-way ANOVA. For the treatment was significant (*P* < 0.05), Tukey’s multiple-comparison tests were used to analyze significant differences. Different letters above the columns indicate significant differences at *p* < 0.05 level

Dimorphic transition of *U. esculenta* from haploid yeast to dikaryotic filaments contains sequential phases [25]. The first typical phenotype is conjugation tubes formation, which is always regarded as the beginning of the dimorphic switching. The two heterogametic cells fused quickly through conjugation tubes and then dikaryotic filaments formed and elongated. At last, aerial hyphae extended, appeared a fuzzy appearance. Under normal mating conditions (on nutrient-rich YEPS medium), the two heterogametic strains UeT14 and UeT55 formed conjugation tubes within 12 h, and then mated to form long hyphae with vacancies. The aerial hyphae could be seen after 24 h, showing a white fuzzy growth (Additional file 2: Figure S1A; [25]). Hyphal length was shorter when arginine added to the YEPS medium, whereas no significant change was observed after the addition of metabolic products of arginine (urea or ornithine) (Fig. 1). Notably, higher concentration of arginine exhibited a stronger inhibition on hyphal length at 3 days after mating assays (Fig. 1). We also examined the dimorphic transition process after 10 mM arginine added under both nutrient-rich condition (YEPS medium) and nutrient-poor condition (BM medium). When mating on BM medium, dimorphic switching began after nearly 24 h cultured, a 12 h delay compared to that on YEPS medium. After 10 mM arginine added, a small amount of conjugation tubes formed at 24 h under nutrient-rich condition or at 36 h under nutrient-poor condition (Table 1), indicating a ~ 12 h delay in dimorphic switching caused by 10 mM exogenous arginine.

**Table 1:** The conjugation formation conditions of WT strains or *UeArginase* mutations on YESP/YEPS-ARG/BM/BM-ARG medium after mating

Strains	Type of medium	Time of conjugation tubes formation (hr)	Amount of the conjugation tubes
WT	YEPS	12	0.8 ± 1.2%
	YEPS-ARG	24	16.2 ± 2.1%
	BM	24	6.9 ± 1.3%
	BM-ARG	36	20.6 ± 3.5%
*UeArginase* mutants	YEPS	36	6.7 ± 2.2%
	YEPS-ARG	48	22.5 ± 4.2%
	BM	48	12.9 ± 3.3%
	BM-ARG	60	20.7 ± 1.7%

The conjugation formation conditions of WT strains or *UeArginase* mutations on YESP/YEPS-ARG/BM/BM-ARG medium after mating

### *UeArginase* is required for arginine metabolism

**Fig. 2:**
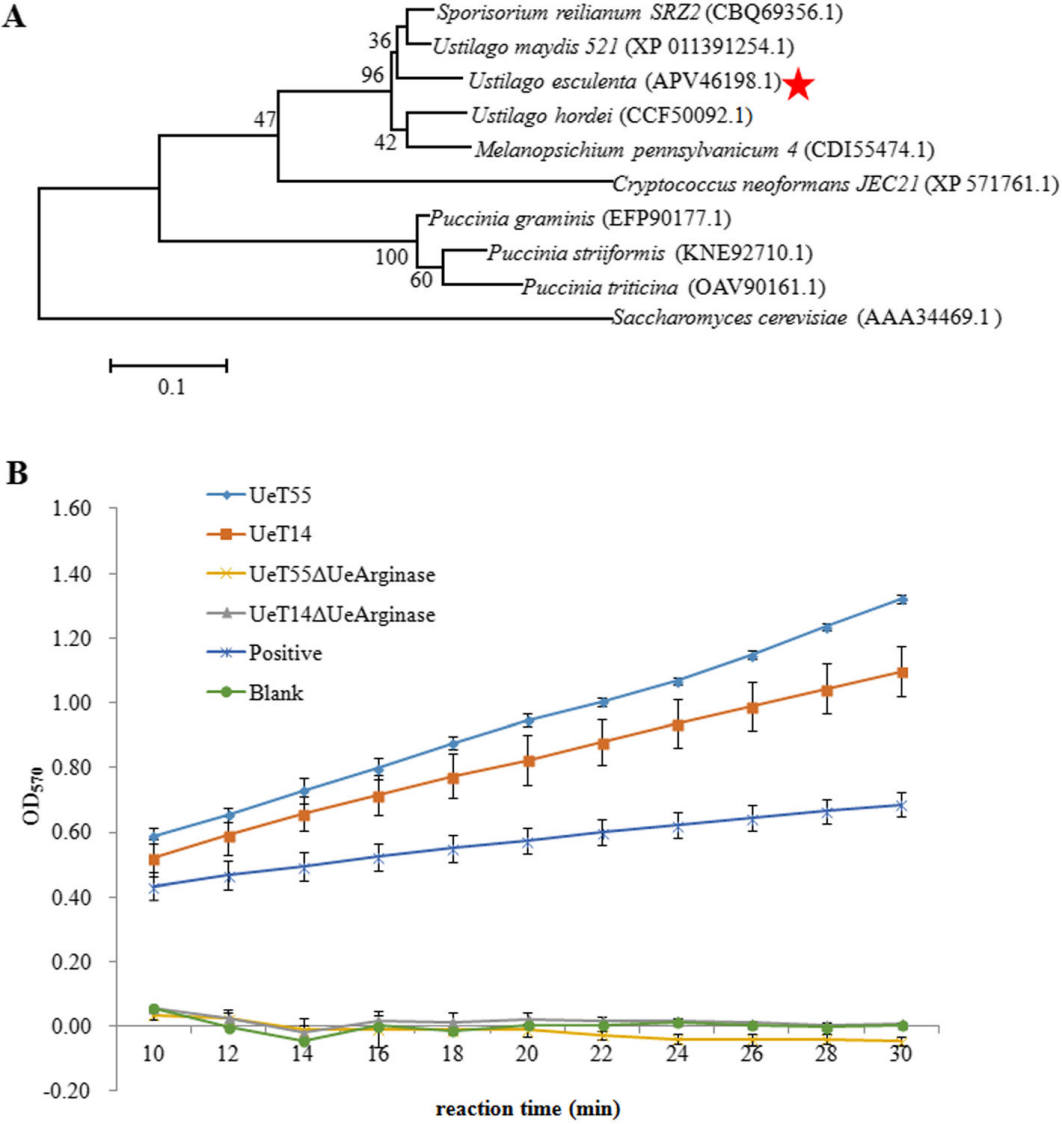
Characterized of UeArginase. **a** Phylogenetic analysis of UeArginase. Sequence alignment was performed using ClustalW program and phylogenic tree was constructed using the neighbor-joining method. **b** Arginase activity was completely lost in when *UeArginase* was deleted. The values of OD_570_ in Arginase positive control increase gradually with the increase of reaction time. Greater changes of the values of OD_570_ in the same reaction time interval indicated a higher Arginase activity. The reaction curve of any samples similar to Blank means no Arginase activity

Based on the whole genome shotgun sequencing (TLW00000000, 21], genes in arginine synthetic and metabolic pathway of *U. esculenta* were predicted by BlastP searching using the protein sequences of *U. maydis* [29] (Additional file 1: Table S2). At the beginning of dimorphic switching, we found that only *g6606* was higher expressed in strains cultured on YESP-ARG medium, comparing to that cultured on YEPS medium (Additional file 3: Figure S2). Then, the genomic sequence and CDS sequence of *g6606* in *U. esculenta* were cloned (APV46198.1). It contains an open reading frame with 957 nucleotides encoding a polypeptide of 319 amino acids. Multiple sequence alignment showed that its identity to Arginase in *U. maydis* and *Sporisorium reilianum* is more than 90% (Fig. 2a, Additional file 4: Figure S3). The g6606 deletion mutants were generated from WT strains (UeT14 and UeT55) using homologous recombination. Its deletion result in completely loss of arginase activity (Fig. 2b). So it is named *UeArginase*.

**Fig. 3:**
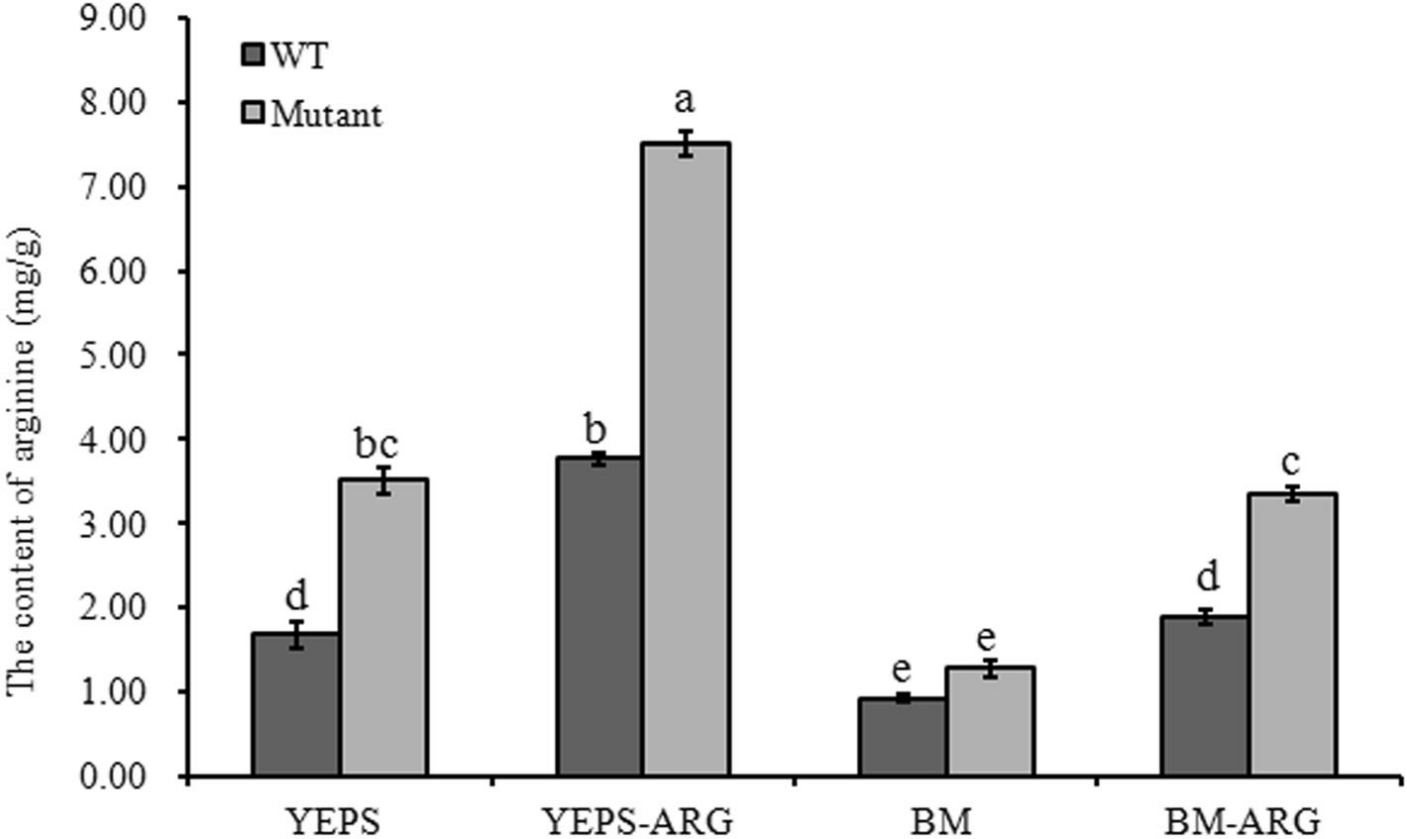
The content of arginine in haploid strains of wide types or *UeArginase* mutations after 12 h liquid culturing. YEPS and BM on the X-axis represent different liquid media. -ARG represent 10 mM arginine added to specific medium. Differences in the content of arginine were analyzed using the generalized linear model (GLM) with the variables of medium, the amount of arginine added and strains, and 3 blocks. For the interaction effect of the three variables was significant (*P* < 0.05), Least squares means were computed for multiple-comparison. Different letters above the columns indicate significant differences at *p* < 0.05 (Tukey)

To further clarify the role of *UeArginase* in arginine metabolism, the changes of arginine content were tested in *UeArginase-*deletion mutant. According to the result of HPLC test, the content of arginine was ~ 1.7 mg/g in WT strains, remarkably lower than that ~ 3.5 mg/g in mutants, after 12 h liquid culturing in YEPS medium. When 10 mM exogenous arginine was added to the YEPS liquid medium, the content of arginine was obviously increased both in WT strains and mutants and *UeArginase* mutants. However, it reached ~ 7.5 mg/g in mutants, 3.5 mg/g more than that in WT strains (Fig. 3). Additionally, the effect of *UeArginase* on the metabolism of arginine under nutrient-poor conditions was assessed., the arginine content was not significantly different between WT strains (~ 0.9 mg/g) and *UeArginase* mutants (~ 1.3 mg/g) after liquid culturing in BM mudium. When 10 mM exogenous arginine added to BM medium, the *UeArginase* mutants showed an obvious defect in arginine metabolism, that the content of arginine increased by 2 mg/g in mutants, whereas that only increased by 1 mg/g in WT strains (Fig. 3). All the data suggest that *UeArginase* is required for arginine metabolism.

### *UeArginase* mutation affects the beginning of dimorphic transition

**Fig. 4:**
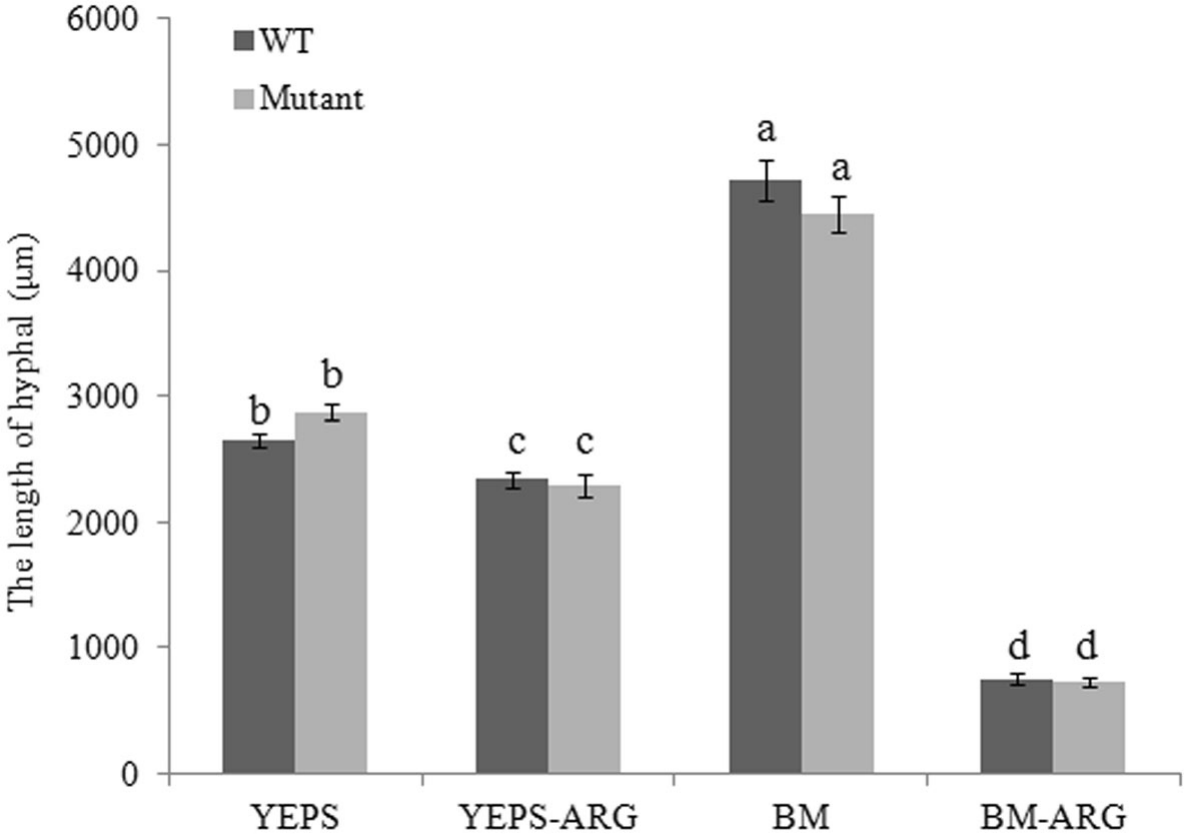
The hyphal growth of WT strains or *UeArginase* mutations after mating. Morphology of colonies were photographed (a) and hyphal length were measured (b) under stereomicroscope at 3 days after conjugation tubes formation during mating assays carried out. Scale bar (in a) represents 1500 μm. YEPS and BM on the X-axis (in b) represent different liquid media. -ARG represent 10 mM arginine added to specific medium. Differences in the hyphal length were analyzed using the generalized linear model (GLM) with the variables of medium, the amount of arginine added and strains, and 3 blocks. For the interaction effect of the three variables was significant (*P* < 0.05), Least squares means were computed for multiple-comparison. Different letters above the columns indicate significant differences at *p* < 0.05 (Tukey)

In order to detect the role of arginine metabolism in dimorphic transition, an interval 12 h observation during the mating process of *UeArginase* mutants was carried out. Results showed that dimorphic switching of the mutants was delayed nearly 24 h when comparing that of the WT strains, both under nutrient-rich and nutrient-poor conditions (Table 1). In more details, during mating assays, we could see the conjugation tubes and fused cells of mutants at 36 h on YEPS medium and at 48 h on BM medium, while they appeared in WT strains at 12 h on YEPS medium and 24 h on BM medium (Additional file 2: Figure S1A, C, E, G). Similar to the WT strains, these mutants mated 12 h later on the medium with 10 mM exogenous arginine than on the medium without arginine (Table 1; Additional file 2: Figure S1). Furthermore, hyphal length was measured separately at 3 days after conjugation tubes were observed. No fuzzy growth was observed when cultured on BM medium (Fig. 4a). Howerer, the length of dikaryotic filaments of either WT strains or mutants formed on BM medium was more than 4000 μm, nearly 2 times longer than that on YEPS medium. Besides, hyphal length of either WT strains or mutants was shorted to ~ 700 μm when mated on BM-ARG medium (Fig. 4b). Even more, the WT strains and mutants appeared white fuzzy growth and no obvious difference in hyphal density and length no matter cultured on YEPS medium or YEPS-ARG medium (Fig. 4b). All the data indicated that *UeArginase* mutation did not affect the hyphal growth.

### *UeArginase* mutation and **exogenous arginine** attenuates PKA-mediate signaling

**Fig. 5:**
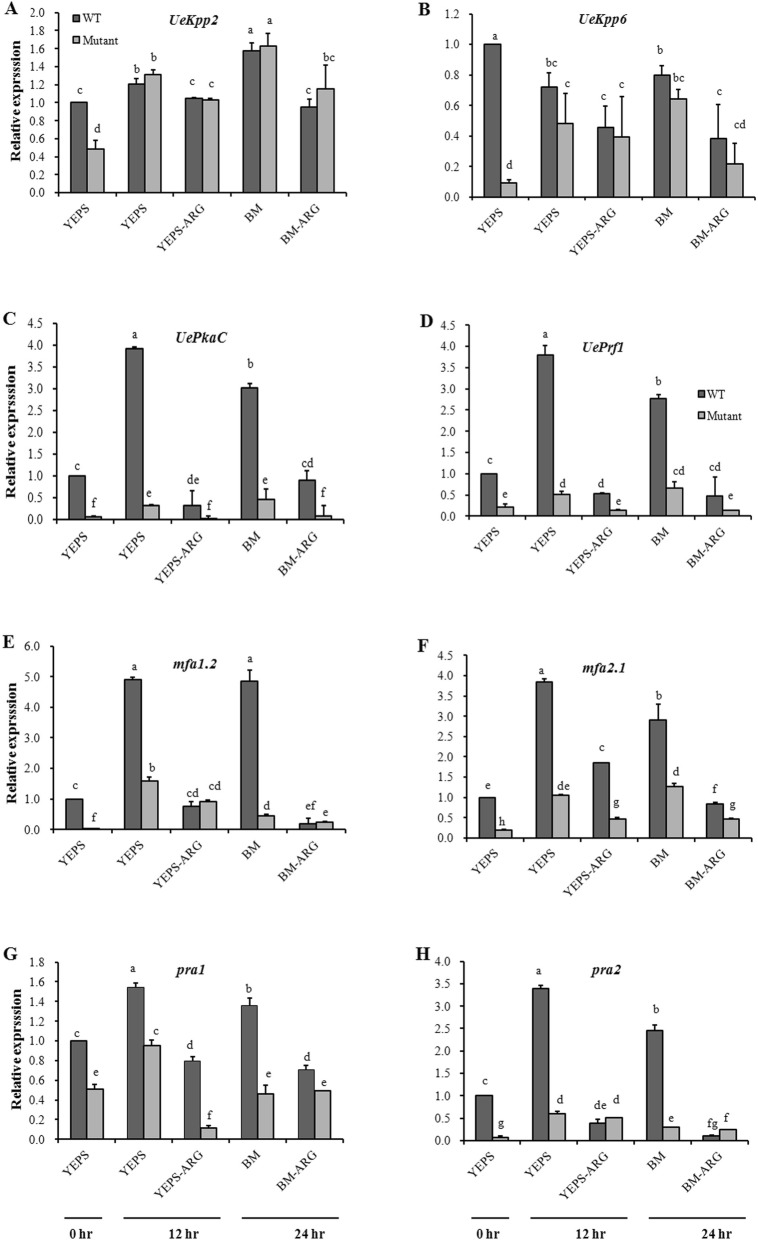
Relative expression of genes in PKA and MAPK pathway and *a* mating type genes during mating process. The basic expression of *UeKpp2* (**a**), *UeKpp6* (**b**), *UePkaC* (**c**), *UePrf1* (**d**), *mfa1.2* (**e**), *mfa2.1* (**f**), *pra1* (**g**) and *pra2* (**h**) in WT strains and *UeArginase* mutations at 0 h and 12 h after mating on YESP/YEPS-ARG medium or 24 h after mating on BM/BM-ARG medium during mating procedure. At least 5 individual colonies were collected in the mating assay. The haploid samples mixed at the beginning of mating assays were collected as the tested samples of 0 h. The mixed wild type strains collected at 0 h on YEPS medium was used as a contrast to evaluate the relative expression of genes during mating procedure. Differences in the gene expression levels were analyzed using the generalized linear model (GLM) with the variables of the stage life, medium, the amount of arginine added and strains, and 3 blocks. For the interaction effect of the four variables was significant (*P* < 0.05), Least squares means were computed for comultiple-comparison. Different letters above the columns indicate significant differences at *p* < 0.05 (Tukey)

In order to explore whether the MAPK and PKA signaling pathways, proved at the heart of dimorphic transition network [14], were affected by the *UeArginase* mutation during mating procedures, we further compared the expression levels of genes involved in the MAPK and PKA pathway. WT strains and mutants were tested during the mating procedure on YEPS, YEPS-ARG, BM or BM-ARG medium. *UeKpp2* (KU855052) and *UeKpp6* (KU855053) in MAPK pathway, *UePkaC* (KU302685) in PKA pathway and *UePrf1* (KT343766) downstream of MAPK and PKA pathway were chosen. In the haploid strains (samples of 0 h in mating assays), the expression levels of *UeKpp2*, *UeKpp6*, *UePkaC* and *UePrf1* were significant lower in mutants than those in WT strains, indicating a reduced basal expression of them in *UeArginase* mutants (Fig. 5a-d). After 12 h mating on YESP medium or 24 h mating on BM medium (conjugation tubes were formed, indicating a dimorphic transition happened), *UePrf1* and *UePkaC* were up-regulated both in WT strains and mutants as compared with those before mating (0 h after mating), but their expressions did not reached normal levels in mutants, less than a third of those in WT strains (Fig. 5a, b). Besides, *UePrf1* and *UePkaC* in 10 mM exogenous arginine treated samples were not induced after mating at 12 h on YESP medium or 24 h on BM medium (Fig. 5a, b). However, the expression levels of *UeKpp2* and *UeKpp6* were similar between WT strains and mutants after mating either in YEPS medium or BM medium, whether or not treated with exogenous arginine (Fig. 5c, d). Furthermore, the pheromone response related *a* genes, which have been proved to be regulated by *PkaC* and *Prf1* [30, 31], were also analyzed. The functional pheromone genes *mfa1.2* (KT343772) in UeT14 and *mfa2.1* (KT343776) in UeT55, and the pheromone response genes *pra1* (KT343774) in UeT14 and *pra2* (KT343777) in UeT55 were chosen. In the haploid strains, they showed significantly lower expression levels in mutants than those in WT strains (Fig. 5e-h). During mating, all the *a* genes were up-regulated either in mutants or WT strains both in YEPS and BM medium, but significantly lower expression level was found in mutants than those in WT strains (Fig. 5e-h). In addition, 10 mM exogenous arginine adding to the mating medium also inhibited *a* genes expression after mating when compared to that mated at 12 h on YESP medium or 24 h on BM medium.

## Discussion

In this study, we have elucidated the fact that arginine may function on the dimorphic transition of *U. esculenta*. In contrast to an inducer of hyphae formation in several fungi such as typical opportunistic fungal pathogen *C. albicans* or typical plant pathogen *C. ulmi* [8, 9], higher concentrations of arginine required for vegetative growth is negative for dimorphic transition in the endophytic-like fungus *U. esculenta*, in which the transition is nonreversible.

The inhibitory effect of arginine on dimorphic transition was first observed in N-source bias test of *U. esculenta* [25]. As known to all, the lack of N sources facilitates the formation of pseudohypha or dimorphic switching in most fungi [32]. In this study, results showed that when *U. esculenta* cultured on nutritious poor medium (BM), the dimorphic switching was delayed by 12 h (Fig. 4a), but the hyphal length was more than 1.5 times longer, comparing to that cultured on nutritious rich medium (YESP) (Fig. 4b). According to the fact that the growth rate of *U. esculenta* in BM medium is slow [25], we thought that the lack of available N sources indeed promoted the elongation of mycelium, while the slowness of dimorphic transition on BM medium may be related to the cell density which is an important factor in dimorphism in many fungi [32]. When exogenous arginine added to BM or YEPS medium, the growth rate of *U. esculenta* was not influenced (Additional file 5: Figure S4), but a 12 h delay of dimorphic transition happened (Table 1). This was indicated exogenous arginine indeed influenced dimorphic transition. Similar findings was discovered previously that arginine and its precusor and metabolic products play important roles in fungal dimorphism [9, 14].

In this study, we firstly confirmed that only exogenous arginine inhibited dimorphism of *U. esculenta*, but two critical arginine anabolites ornithine and urea did not (Fig. 1, Table 1). What’s more, this inhibition is proportional to the concentration of arginine (Fig. 1). Meanwhile, only *UeArginase* from all the arginine biosynthetic and metabolic pathway genes showed a higher expression in response to excessive exogenous arginine (Additional file 3: Figure S2). Besides, the expression of *UeArginase* was decreased during mating procedure (Fig. 7). Therefore, we wonder whether the arginine metabolic pathway plays the critical role in the dimorphism of *U. esculenta*. However, during mating process, *UeArginase* deletion mutants had no obviously different in hyphal length when compared with WT strains on the same medium (Fig. 4a). What caught our attention was that either deletion of *UeArginase* or exogenous arginine treatment led to increase content of arginine and delayed dimorphic transition (Fig. 4a, Table 1). Previous studies reported that excessive arginine would be stored in the vacuoles and additional arginine assimilation would induce synthetic and metabolic signals communicating between the cytosol and mitochondrial matrix and storage [33, 34]. So, we speculated that arginine itself was the influence factor and its dynamic equilibrium is critical for dimorphic transition of *U. esculenta*.

**Fig. 6:**
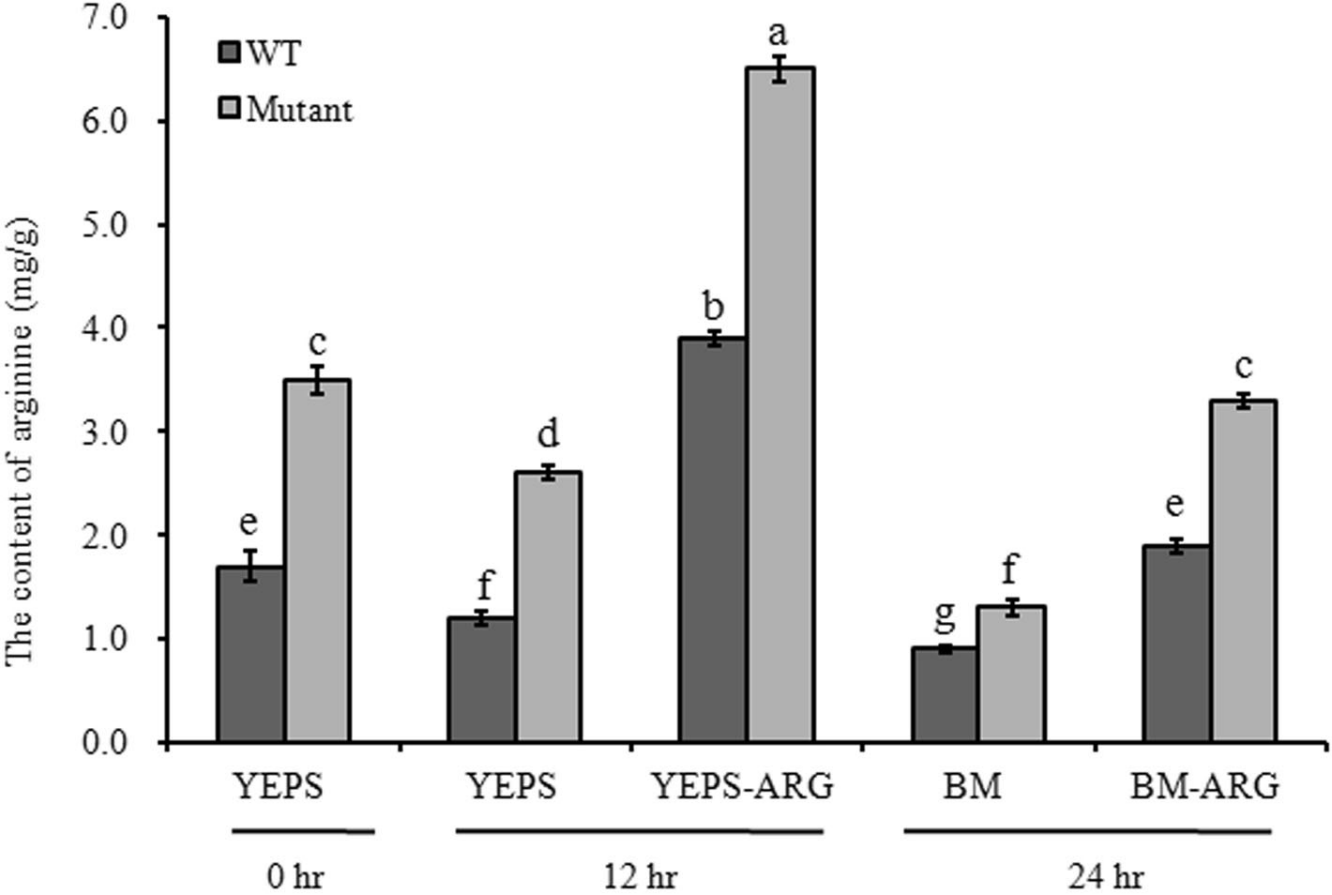
The content of arginine in WT strains or *UeArginase* mutations during mating process. The contents of arginine in WT strains and *UeArginase* mutations were measured at 0 h and 12 h after mating on YESP/YEPS-ARG medium or 24 h after mating on BM/BM-ARG medium during mating procedure. At least 5 individual colonies were collected in the mating assay. The haploid samples mixed at the beginning of mating assays were collected as the tested samples of 0 h. Differences in the content of arginine were analyzed using the generalized linear model (GLM) with the variables of the stage life, medium, the amount of arginine added and strains, and 3 blocks. For the interaction effect of the four variables was significant (*P* < 0.05), Least squares means were computed for comultiple-comparison. Different letters above the columns indicate significant differences at *p* < 0.05 (Tukey)

**Fig. 7:**
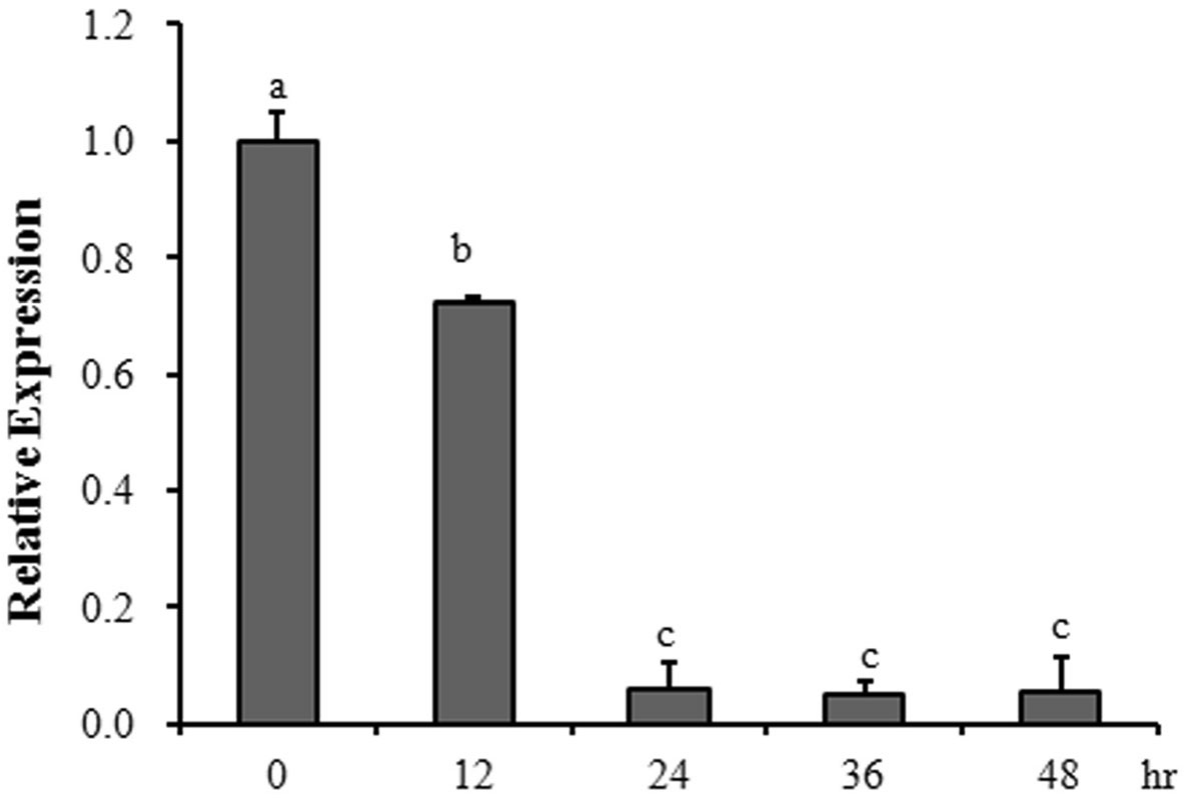
Relative expression of *UeArginase* during mating process. At least 5 individual colonies were collected every 12 h in the mating assay until 48 h. The haploid samples mixed in the beginning of mating assays were collected as the tested samples of 0 h, which was used as a contrast to evaluate the relative expression of *UeArginase* during mating procedure. Differences in gene expression levels were analyzed by One-way ANOVA. For the treatment was significant (*P* < 0.05), Tukey’s multiple-comparison tests were used to analyze significant differences. Different letters above the columns indicate significant differences at *p* < 0.05 level

Furthermore, the content of endogenous arginine in WT strains and mutants during their mating procedure were detected by HPLC when cultured on YEPS, YEPS-ARG, BM or BM-ARG medium. Results showed a decrease of endogenous arginine appeared both in WT strains and mutants 12 h after mating on YESP or 24 h after mating on BM medium (Fig. 5; from ~ 1.7 mg/g to ~ 1.2 mg/g on YEPS medium and to ~ 0.9 mg/g on BM medium in WT strains; from ~ 3.5 mg/g to ~ 2.6 mg/g on YEPS medium and to ~ 1.3 mg/g on BM medium in mutants) (Fig. 6). The results indicated a decreased endogenous arginine synthesis or an increased endogenous arginine metabolism happened during dimorphic transition. Besides, the significantly reduced expression of *UeArginase* (Fig. 7) implicating the endogenous arginine metabolism was inhibited during mating procedure. So, a decreased endogenous synthesis may be necessary during mating. As we known, the MAPK and PKA signal pathways and *a* genes were implicated in dimorphic transition [1, 26]. *UeKpp2*, *UeKpp6*, *UePkaC*, *UePrf1* are key genes in these two signal pathways in *U. esculenta* [27, 28]. Results showed a reduced basal expression of *UeKpp2*, *UeKpp6*, *UePkaC*, *UePrf1* and *a* genes in mutants than those in WT strains, and a persistent lower expression levels of *UePkaC*, *UePrf1* and *a* genes in mutants compared to WT strains when mating on medium without arginine. Also, persistent lower expression levels of *UePkaC*, *UePrf1* and *a* genes appeared in strains mating on medium with arginine compared to that on medium without arginine (Fig. 5c-h).

All the data indicated that the content of endogenous arginine may be negatively correlated to the dimorphic transition and the expression of *UePrf1*, *UePkaC* and *a* genes. These results cleared that arginine, not its metabolic products, is an inhibitor for dimorphism of *U. esculenta*, by inhibiting PKA pathway. But how arginine acts on PKA signal pathway to regulate dimorphism transition in *U. esculenta* still need much more further study.

## Conclusion

In this study, we explored whether the arginine or its metabolic pathway plays the inhibition effect on the dimorphism of *U. esculenta*. Results showed that the exogenous arginine or *UeArginase* mutants slowed down the dimorphic transition of *U. esculenta*, along with increased content of endogenous arginine and down regulated expressions of *UePkaC*, *UePrf1* and *a* genes during mating process. It is speculated that arginine itself has a direct inhibition on the dimorphic transition of *U. esculenta*, related to its own concentration, independent of its hydrolysis by UeArginase.

## Methods

### Strains and growth conditions

*U. esculenta* wild type (WT) strains UeT14 (a1b1) and UeT55 (a2b2) [25] isolated from grey Jiaobai of the cultivar Longjiao 2# (Variety number: 2,008,024 in vegetable of Zhejiang Province) in Tongxiang (30°68′87.82 N, 120°54′05.49E) were used in this study. All the strains were cultured at 28 °C on YEPS medium (yeast extract 10 g/L, peptone 20 g/L, sucrose 20 g/L).

### Gene cloning and bioinformatics analysis

DNA was extracted using CTAB method [35]. Total RNA was extracted by Spin Column Fungal Total RNA Purification Kit (B518659, Sangon Biotech, China). cDNA was synthesized by PrimeScript™ II 1st strand cDNA Synthesis Kit (6210A, Takara, Japan). The genomic sequence of *UeArginase* was identified by PCR-sequencing using the primers Arginase-gF/gR (Additional file 1: Table S1). The open reading frame (ORF) of *arginase* was amplified by RT-PCR with the primers Arginase-cF/cR (Additional file 1: Table S1). The intron of arginase was verified through comparing the genome sequence and ORF of *UeArginase* by Clone Manger program*.* Multiple amino sequence alignment was performed by DNAMAN with clustalW methods. Phylogenetic tree of UeArginase in *U. esculenta* and related species was constructed with the MEGA 5 programs using the neighbor-joining method.

### Arginase activity assay

1 mL haploid strains liquid cultured with an OD_600_ of 0.5 was harvest and washed by cold PBS twice. Cells were resuspended in 100 μL of ice cold Assay Buffer on ice and transferred it to 1.5 mL tubes after grinding broken. At last, the supernatant was collected after centrifuge for 5 min at 4 °C at 10,000 x g, and transfered to a clean tube for Arginase activity assays, according to the operation manual of Arginase Activity Assay Kit (Colorimetric, ab180877, Abcam).

### *UeArginase* deletion strains construction

For generation of stable transformants, Hygromycin B was chosen as the selection maker and homologous recombination strategy was introduced [36, 37]. ~ 1 kb fragments of the upstream and downstream of the open reading frame of *UeArginase* were amplified with the primers Arginase-UF1/UR1 and arginase-DF2/DR2, respectively. The hygromycin resistance gene including the promoter and terminator sequence was cloned, dividing into two fragments (one containing 5′ sequence was amplified by paired primers Hyg-F/Hyg3-R and the other one containing 3′ sequence was amplified by Hyg4-F/Hyg-R with a 25 bp overlapping sequence). Two fragments amplified by Arginase-UF1/UR1 and Hyg-F/Hyg3-R were linked by two rounds of fusion PCR to generated the linear upstream fragment for transformation. The linear downstream fragment for transformation was generated by fusions PCR of the two fragments amplified by Hyg4-F/Hyg-R and Arginase-DF2/DR2. The two constructed linear fusion fragments were used to generate *UeArginase* mutant by PEG-mediated protoplast transformation [37]. The candidate transformants would be obtained after the plate incubated at 28 °C on regeneration medium [37] for 5–7 days and selected by normal PCR and confirmed by qRT-PCR and southern blot [27]. All the primers used were listed in Additional file 1: Table S1.

### Mating tests

Colony cultured strains were expanding cultured in liquid YEPS medium with a final values of OD_600_ around 1.0, then collected by centrifugation and resuspended in liquid YEPS medium to an OD_600_ of ~ 1.8. Sexual compatible strains were mixed with same volume and then spotted on solid plates of test medium. Plates were sealed with parafilms and cultured at 28 °C. Samples collection, observation and pictures capture under microscopy were carried out every 12 h over 3 days. XD Series Biological Microscope (XD30, SUNNY, China) is for observation of cell structure, such as the morphology of yeast cells, the conjugation tube and filament. SZN Zoom Stereo Microscope (EX31, SUNNY, China) is mainly used to observe the morphology of colony. Hyphal growth was observed under SZN Zoom Stereo Microscope to evaluate the mating response. Test medium prepared as follows: Basic medium (BM), including K_2_HPO_4_ 1 g/L, MgSO_4_·7H_2_O 0.5 g/L, FeSO_4_·7H_2_O 0.01 g/L and KCl 0.5 g/L, autoclaved for sterilization, then adding 20 mmol/L KNO_3_ and 50 mmol/L sucrose which was filtered by Millipore filters (0.22 μm); YEPS-ARG medium, including yeast extract 10 g/L, peptone 20 g/L, sucrose 20 g/L, Agar 15 g/L, autoclaved 15 min, then adding filtered L-Arginine 2 g/L; BM-ARG medium, including K_2_HPO_4_ 1 g/L, MgSO_4_·7H_2_O 0.5 g/L, FeSO_4_·7H_2_O 0.01 g/L and KCl 0.5 g/L, autoclaved for sterilization, then adding 20 mmol/L KNO_3_, 50 mmol/L sucrose and 2 g/L L-Arginine which were filtered by Millipore filters (0.22 μm) [25].

### HPLC assay

The content of endogenous arginine was detected by HPLC assay. Samples of mating assays were selected based on their status and hydrolyzed in hydrochloric acid for 22 h (0.1 g in 1 mL hydrochloric acid). Then 50 °C N_2_ was used to dry all the samples. The residue dissolved in 1 ml hydrochloric acid and filtrated by 0.22 μm filter membrane. 20 μl sample solution was detected under 570 nm and 440 nm channel of #2622 PH columm (4.6 mm*60 mm, 3 μm) in HPLC system (L-8900 Hitachi, Japan). Read the characteristic peak area at 28.667 min. Then put it into formula X_arg_ $=\frac{C\ast 174.2\ast V\ast n\ast 100}{\mathrm{m}\times {10}^6}$ (X_ar_ means the content of arginine in 100 g sample, C means the concentration of arginine which could be read through the chromatogram, V means the volume of hydrolysable arginine, n means dilution ratio, m means the mass of hydrolysable arginine).

### Quantitative real-time PCR analysis

Gene expression was evaluated by real-time PCR. Samples of mating assays were selected based on their status during mating on different medium. Cells were scraped from the plates to extract RNA. The PrimeScript™ RT reagent Kit with gDNA Eraser (Perfect Real Time, RR047A, TAKARA, Janpan) was employed for cDNA synthesis. qRT-PCR was performed on a Bio-Rad (CFX Connect™ Real-Time System, Bio-Rad, USA) using the SYBR Premix Ex Taq™ (Tli RHaseH Plus, RR420A, TAKARA). The parameters and progresses were as follows: initial denaturation 95°Cfor 30 s, followed by 40 cycles of amplification (95 °C for 15 s, 58 °C for 30 s), and a melting curve at the end of each reaction consisting of a cycle (95 °C for 15 s, 60 °C for 1 min) and a slow temperature increase to 95 °C at the rate of 0.3 °Cs^− 1^. The comparative CT (2^- △△CT^) method was used for calculating the relative gene expression [38]. *β-Actin* was used as the internal reference. The primer sequences were listed in Additional file 1: Table S1.

### Statistical analysis

All experiments were performed in triplicate and data were shown as mean ± SEM from three independent experiments. All data from three independent experiments were analyzed according to the method of Student’s t-test or ANOVA or generalized linear model, and *P* < 0.05 was considered to indicate statistical significance.

## Additional files

Additional file 1: **Table S1.** Primers used in this study. **Table S2.** Predicted genes in arginine synthesis and metabolic pathway. (DOCX 18 kb)

Additional file 2: **Figure S1.** Morphology of colonies and cells of WT strains or *UeArginase* mutations during mating process. The WT strains were spotted alone on YEPS (a), YEPS-ARG (b), BM (c), BM-ARG (d) plates and UeArginase mutations were spotted alone on YEPS (e), YEPS-ARG (f), BM (g), BM-ARG (h) plates. Tracing observation every 12 h during mating procedure was carried out until 48 h. Typical cells morphology of indicated strains during mating was represented in the top left corner of the image of colony morphology. The scale is in the lower right corner of each image. (JPG 2360 kb)

Additional file 3: **Figure S2.** Relative expression of genes in the arginine synthesis and metabolic pathway. At least 5 individual colonies were collected at 12 h after mating. The samples on YEPS medium was used as a contrast to evaluate the relative expression of *UeArginase* during mating procedure. Differences in gene expression levels between strains cultivated in YESP/YEPS-ARG medium were analyzed by Student’s t-test. Pentagonal stars above the column indicate a significant difference from others at *p* < 0.05 level. (JEPG 320 kb) (JPG 319 kb)

Additional file 4: **Figure S3.** Amino acid sequence alignment of UeArginase. The multiple sequence alignment was performed by DNAMAN. Blue highlighted amino acids represent 100% identity between UePrf1 and other sequences, while green highlighted amino acids represent 75%. (JPG 2909 kb)

Additional file 5: **Figure S4.** Comparable growth rate of UeT14 under BM or YEPS medium after exogenous arginine added. The UeT14 strain were re-suspended to an OD_600_ of 1.0 after liquid cultured. A cell suspension of 1 mL in 100 mL of prepared liquid medium YEPS, YEPS-ARG, BM, BM-ARG. Growth rates of the UeT14 were measured by OD_600_ at an interval of 12 h culture in prepared medium. (JPG 24 kb)

## Data Availability

The datasets used and analysed during the current study available from the corresponding author on reasonable request.

## Abbreviations

BM: Basic medium
cAMP: cyclic adenosine monophosphate
HPLC: High performance liquid chromatography
MAPK: mitogen-activated protein kinase
PKA: cAMP-dependent protein kinase A
WT: Wild type
