# Supplementary material for: Cloning and disruption of the UeArginase in Ustilago esculenta: evidence for a role of arginine in its dimorphic transition
Source: BMC Microbiol. 2019 Sep 5;19:208. doi: 10.1186/s12866-019-1588-2 (PMC6727352; doi:10.1186/s12866-019-1588-2)
Supplement: Supplementary file 1 — Table S1. Primers used in this study. Table S2. Predicted genes in arginine synthesis and metabolic pathway. (DOCX 18 kb) [file 12866_2019_1588_MOESM1_ESM.docx]

**Additional file 1: Table S1**

Primers used in this study.

| Primer | Sequence (5’→ 3') | Purpose |
| --- | --- | --- |
| Arginase-gF | CAAAGTACGTGTGCGCTGAGCCAT | Gene clone |
| Arginase-gR | TGGAAGCATTCGCACGTGACGACT |  |
| Arginase-cF | ATGCTCCACGAACGATTCCT |  |
| Arginase-cR | CTAGAGCAGCGTCTCGCCCA |  |
| Arginase-UF1 | CAGAAGCGTGTCGATCGGAC | Delection mutant construction and verification |
| Arginase-UR1 | AGTTACCACGTTCGGCCATCTAGGCCCACGCACCCGCCATGCA |  |
| Arginase-DF2 | GCTGTCAAACATGAGGCCTGAGT TAGGCTCGTACGCACGGTT |  |
| Arginase-DF2 | CATGTGACGTGTGTTGCTCTG |  |
| Hyg-F | TGGCCGAACGTGGTAACTAC |  |
| Hyg3-R | GGATGCCTCCGCTCGAAGTA |  |
| Hyg4-F | CGTTGCAAGACCTGCCTGAA |  |
| Hyg-R | CTCAGGCCTCATGTTTGACA |  |
| UeArginase-qF | CAAGTATGGCATCGGCAAGG | qRT-PCR |
| UeArginase-qR | TGGGAGCAACGCTGGGGTC |  |
| mfa1.2-QF | TTCCATCTTCACTCAGCACGC |  |
| mfa1.2-QR | AGGCGACAATACATGTGGAG |  |
| pra1-QF | TCCAACCTTGTCATCGCACGAA |  |
| pra1-QR | CGATATGAGTAGATCGATGATG |  |
| mfa2.1-QF | GTTCACTATCTTCGAGACTGTTGC |  |
| mfa2.1-QR | TAGGCCACAACGCAGTAGTTG |  |
| pra2-QF | GTCTTCTCAACATTCAGGCCTGTCT |  |
| pra2-QR | TGAGATAAAATTGTGCAACCGAG |  |
| UePrf1-QF | GAAGCGTTATTGAGCCTGTCG |  |
| UePrf1-QR | TTCTGGGATTGGCACTCTTGTC |  |
| UeKpp6-QF | GCATACTTGCCGAAATGCTCA |  |
| UeKpp6-QR | CGGCTGTAGATGTTGTGGAACT |  |
| β-Actin- QF | CAATGGTTCGGGAATGTGC |  |
| β-Actin- QR | GGGATACTTGAGCGTGAGGA |  |
| UeKpp2-QF | CACCTTGGAAATCCTGGGCA |  |
| UeKpp2-QR | GACGGCGAGAGGATTAGCGTT |  |
| UePkaC-QF | ACGCAAGTTGAGTGGCAGGTA |  |
| UePkaC-QR | AGAACGAACGAGGTGGACGC |  |

**Additional file 1: Table S2**

Predicted genes in arginine synthesis and metabolic pathway

| Predicted genes | Genes ID | Identify with Ustilago maydis |
| --- | --- | --- |
| UeARG1 | g1134 | 99% |
| UeARG2 | g5893 | 75% |
| UeARG3 | g2998 | 95% |
| UeARG4 | g4582 | 98% |
| UeARG5,6 | g880 | 93% |
| UeARG7 | g4051 | 92% |
| UeARG8 | g4718 | 86% |
| UeArginase | g6606 | 95% |
