# Supplementary figures and images for: Cloning and disruption of the UeArginase in Ustilago esculenta: evidence for a role of arginine in its dimorphic transition
Source: BMC Microbiol. 2019 Sep 5;19:208. doi: 10.1186/s12866-019-1588-2 (PMC6727352; doi:10.1186/s12866-019-1588-2)

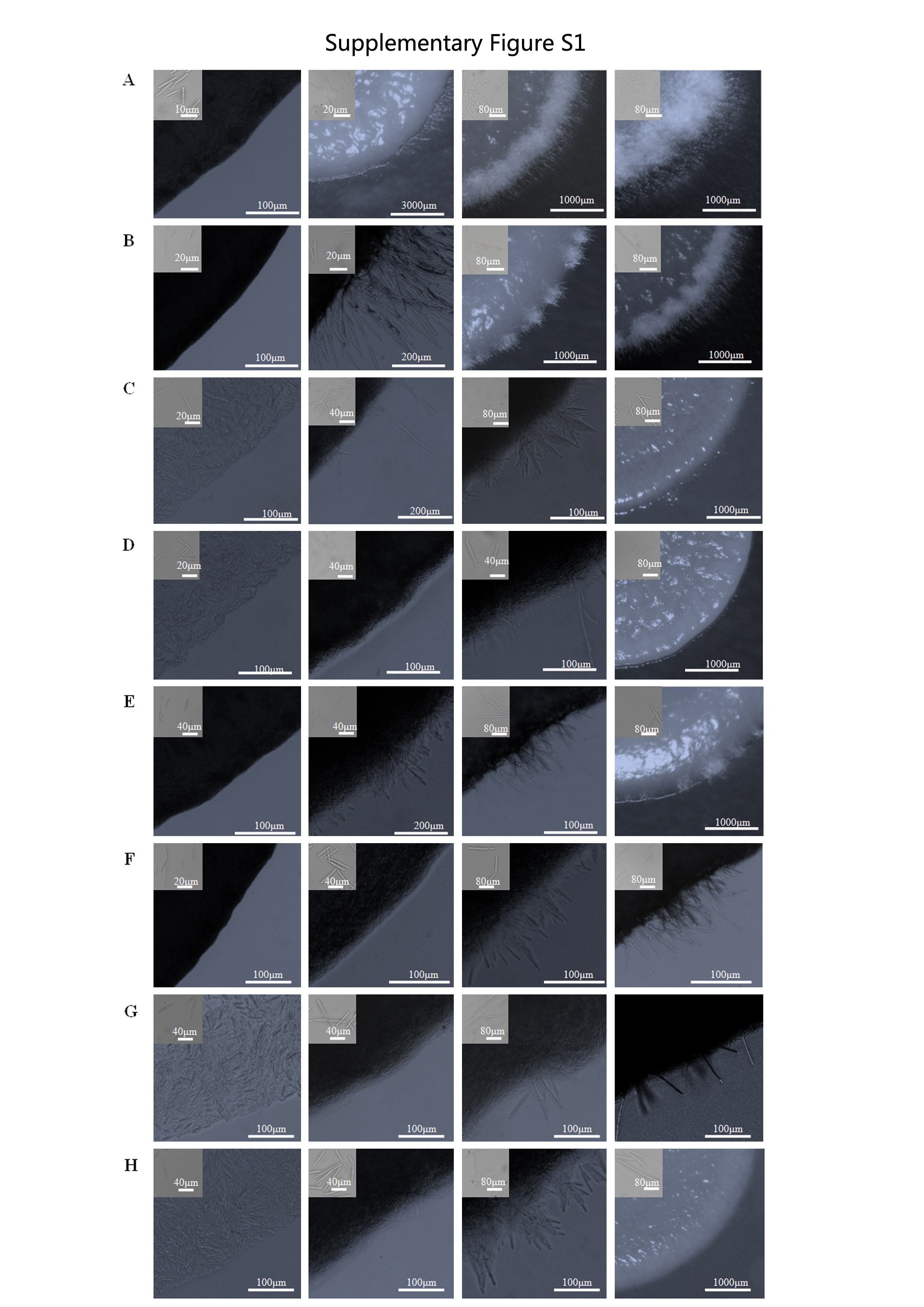

Supplement: Supplementary file 2 — Figure S1. Morphology of colonies and cells of WT strains or UeArginase mutations during mating process. The WT strains were spotted alone on YEPS (a), YEPS-ARG (b), BM (c), BM-ARG (d) plates and UeArginase mutations were spotted alone on YEPS (e), YEPS-ARG (f), BM (g), BM-ARG (h) plates. Tracing observation every 12 h during mating procedure was carried out until 48 h. Typical cells morphology of indicated strains during mating was represented in the top left corner of the image of colony morphology. The scale is in the lower right corner of each image. (JPG 2360 kb) [file 12866_2019_1588_MOESM2_ESM.jpg]

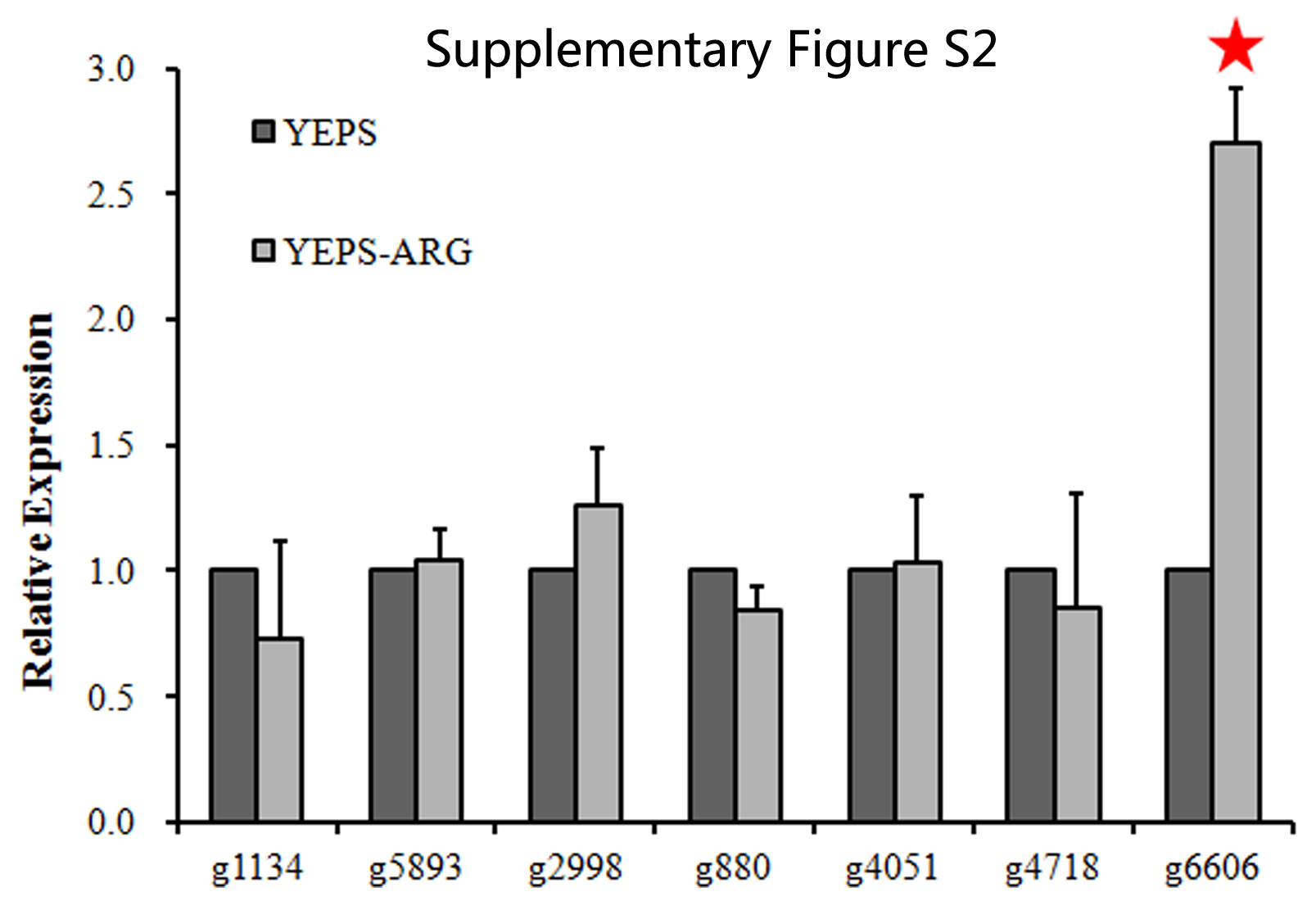

Supplement: Supplementary file 3 — Figure S2. Relative expression of genes in the arginine synthesis and metabolic pathway. At least 5 individual colonies were collected at 12 h after mating. The samples on YEPS medium was used as a contrast to evaluate the relative expression of UeArginase during mating procedure. Differences in gene expression levels between strains cultivated in YESP/YEPS-ARG medium were analyzed by Student’s t-test. Pentagonal stars above the column indicate a significant difference from others at p < 0.05 level. (JEPG 320 kb) (JPG 319 kb) [file 12866_2019_1588_MOESM3_ESM.jpg]

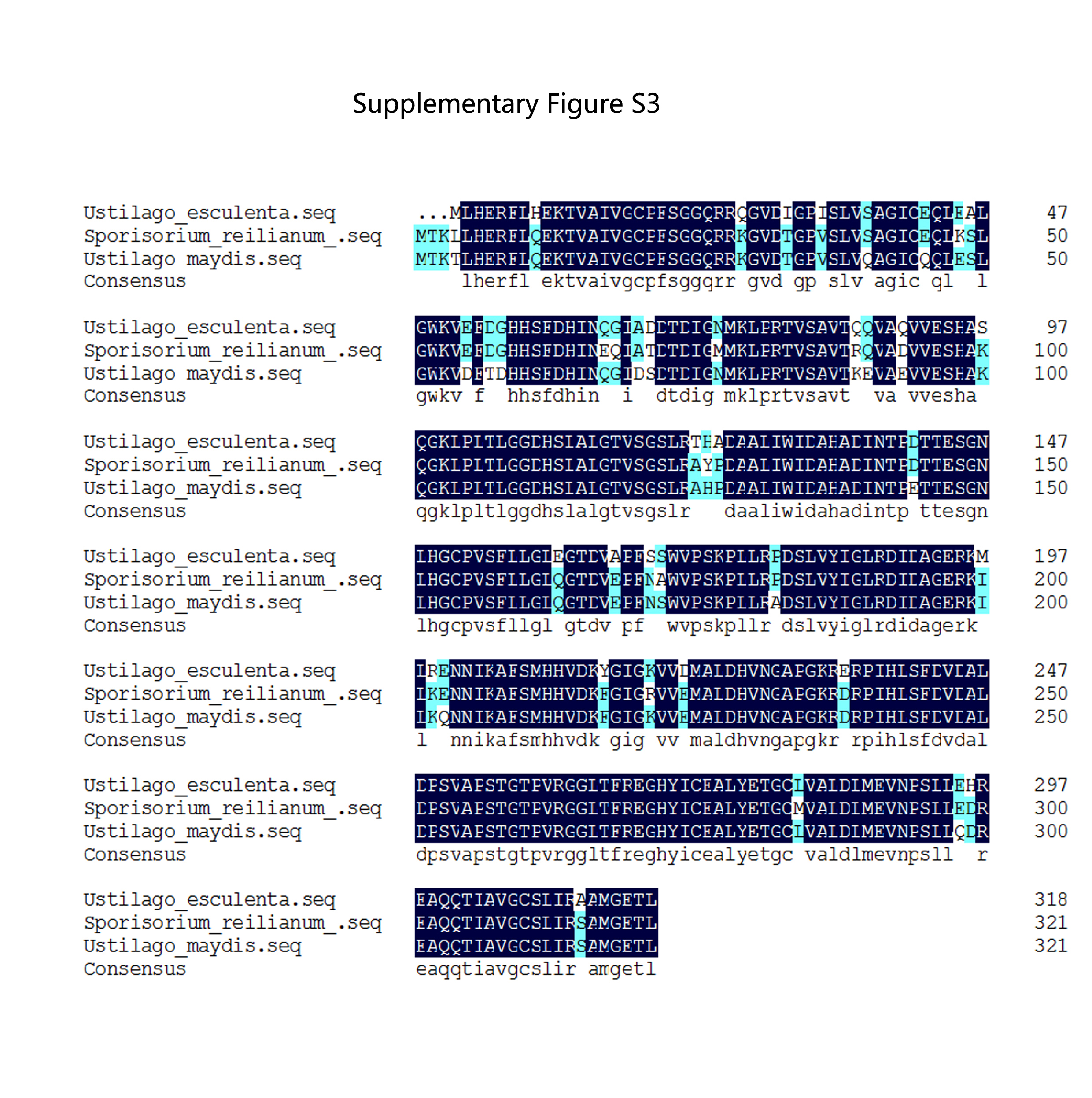

Supplement: Supplementary file 4 — Figure S3. Amino acid sequence alignment of UeArginase. The multiple sequence alignment was performed by DNAMAN. Blue highlighted amino acids represent 100% identity between UePrf1 and other sequences, while green highlighted amino acids represent 75%. (JPG 2909 kb) [file 12866_2019_1588_MOESM4_ESM.jpg]

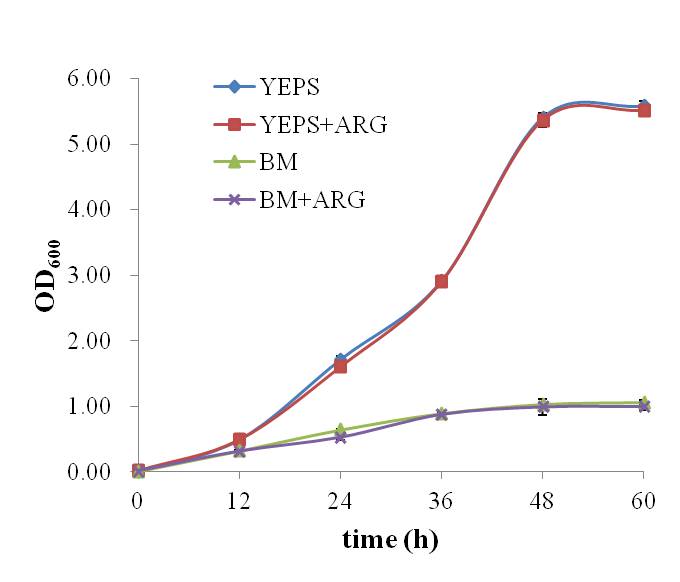

Supplement: Supplementary file 5 — Figure S4. Comparable growth rate of UeT14 under BM or YEPS medium after exogenous arginine added. The UeT14 strain were re-suspended to an OD600 of 1.0 after liquid cultured. A cell suspension of 1 mL in 100 mL of prepared liquid medium YEPS, YEPS-ARG, BM, BM-ARG. Growth rates of the UeT14 were measured by OD600 at an interval of 12 h culture in prepared medium. (JPG 24 kb) [file 12866_2019_1588_MOESM5_ESM.jpg]
